# Genotoxicity Studies of Indole-3-carbinol and *N*-Methoxyindole-3-carbinol—The Effect of Sulphotransferases

**DOI:** 10.3390/ph19060895

**Published:** 2026-06-05

**Authors:** Hansruedi Glatt, Fabian Schumacher

**Affiliations:** Department of Nutritional Toxicology, German Institute of Human Nutrition (DIfE) Potsdam-Rehbrücke, Arthur-Scheunert-Allee 114-116, 14558 Nuthetal, Germany; fabian.schumacher@fu-berlin.de

**Keywords:** N-substituted indole-3-carbinols, glucosinolates, sulphotransferase, SULT1A1, SULT1C4, genotoxicity, DNA adducts

## Abstract

**Background**: Glucosinolates, secondary metabolites present in Brassicales, and their breakdown products have demonstrated various biological effects, including anti-carcinogenic activities in some animal models. The active compounds include indole-3-carbinol (I3C) and *N*-methoxyindole-3-carbinol (NI3C). Building on these findings, several synthetic *N*-substituted I3Cs strongly inhibited the growth of human cancer cell lines. This effect was mediated by reactive intermediates formed by sulphotransferase (SULT) 1A1. **Objective**: We present genotoxicity findings on I3C and N-substituted derivatives, with special consideration given to SULTs. **Methods**: We review genotoxicity findings with I3C, NI3C and their parental glucosinolates. Then, we present some findings hitherto unpublished. **Results**: Neoglucobrassicin and its breakdown product NI3C demonstrated high genotoxic activity in vitro and formed high levels of DNA adducts in animal studies. These effects were strongly enhanced in the presence of SULT1A1. By contrast, glucobrassicin and I3C were weakly mutagenic. New observations include: enhanced activation of NI3C to a mutagen by human SULT1C4 compared to SULT1A1; SULT1A1-dependent genotoxicity of I3C (induction of sister chromatid exchange, SCE); cellular co-localisation of SULT1A1 and DNA adducts formed in the kidneys of NI3C-treated mice. **Conclusions**: I3C and NI3C are genotoxic in the presence of an appropriate SULT, but with large quantitative and qualitative differences (I3C required higher concentrations and induced only SCE, virtually no gene mutations). No information is available regarding the genotoxicity of other *N*-substituted I3C derivatives being developed as antineoplastic drugs. We suspect that they may greatly vary in this activity, which in turn might impact clinical effectiveness as well as adverse side-effects.

## 1. Introduction

**Scheme 1:**
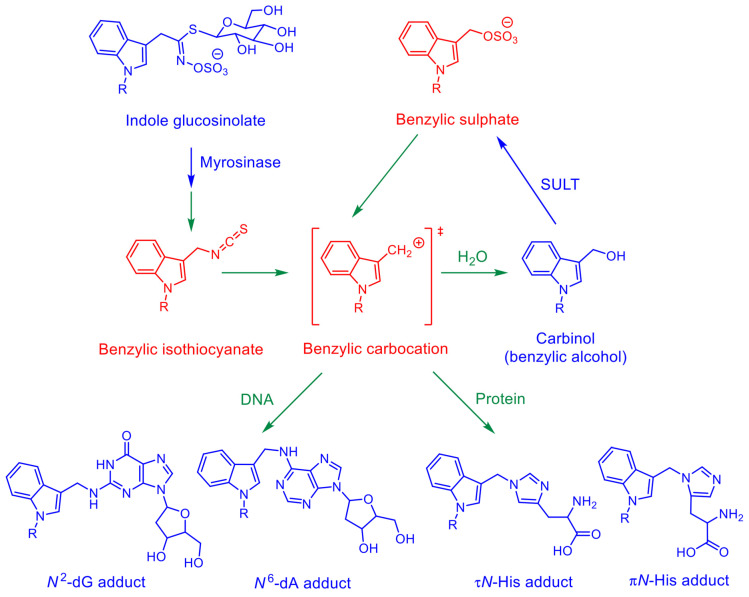
Bioactivation pathways of indole glucosinolates and carbinols to electrophiles forming adducts with DNA and proteins, as demonstrated for neoglucobrassicin (*N*-methoxy-3-indolylmethyl glucosinolate) and *N*-methoxyindole-3-carbinol (R = OCH_3_). The structures of the 2′-deoxyguanosine (dG), 2′-deoxyadenosine (dA), and l-histidine (His) adducts were verified by NMR analyses [43,46]. Adducts with other amino acid residues were also detected in proteins, but their exact structure was not investigated [46]. Analogous activation pathways appear to occur with congeners differing in the substituent (R) at the indole nitrogen. For example, incubation of glucobrassicin (source of I3C present in cruciferous foodstuffs) with DNA in the presence of myrosinase led to the formation of homologous benzylic dG and dA adducts. Various N-substituted I3C derivatives are candidate drugs for treatment of some tumours [34,35]; a congener with a fluorescent residue R was used to demonstrate SULT1A1-dependent covalent binding to proteins in H1299 cancer cells, the putative mechanism underlying growth suppression. Substances marked in blue: chemically relatively stable under physiological conditions (and available as purified compounds); substances marked in red: short-lived, reactive species (not available as chemical standards); blue arrows: enzyme-mediated reactions; green arrows: spontaneous reactions.

Epidemiological studies have found associations between high consumption of *Brassica* vegetables, such as cabbage and broccoli, and decreased risks of cancer at various sites [1,2,3,4,5]. *Brassica* plants (and the order of Brassicales) contain characteristic secondary metabolites, the glucosinolates, which primarily act as a defence against herbivorous animals and microbial infestations [6,7,8,9,10]. This effect requires bioactivation by a β-thioglucosidase; such enzymes (termed myrosinases) are present in the plant in separate compartments and come into contact with the glucosinolates following mechanical damage. The resulting aglycones rearrange into various products, in particular isothiocyanates, the major actual defence chemicals. The isothiocyanates formed from indole glucosinolates are very short-lived; in water they are mainly hydrolysed to carbinols [11,12]. Thus, indole-3-carbinol (I3C) and *N*-methoxyindole-3-carbinol (NI3C) are major breakdown products of glucobrassicin (GBS; Scheme 1, R = H) and neoglucobrassicin (nGBS; Scheme 1, R = OCH_3_), respectively. I3C as well as NI3C lead to strong induction of cytochrome P450 (CYP) enzymes in cells in culture and in vivo, in rat liver, via activation of the arylhydrocarbon receptor (AhR) [13,14,15]. Since only I3C is commercially available, this congener has been used in numerous studies, whereas NI3C has been largely ignored. Dimeric and trimeric condensation products, formed under the acidic conditions of the stomach, were found to be much more potent activators of AhR than the parent I3C [16]. In subcellular hepatic preparations, I3C was oxidised via indole-3-carboxaldehyde to indole-3-carboxylic acid [17]. After oral administration of I3C to mice, the parent compound, indole-3-carboxaldehyde, indole-3-carboxylic acid, and di/trimeric condensation products were detected in blood plasma and various organs [18]. Dietary administration of I3C reduced the incidence, multiplicity, and/or latency of endometrial and mammary gland tumours in various mouse and rat models [19,20,21,22]. It also mitigated tumorigenesis induced in various other organs by benzo[*a*]pyrene (in forestomach) [22], 4-nitroquinoline 1-oxide (in tongue) [23], dimethylnitrosamine (in liver) [24], and tobacco-specific nitrosamines (in lung) [25,26]. In addition, I3C demonstrated antiproliferative effects in cancer cells (e.g., [27,28]). These experimental findings together with the epidemiological observations related to the consumption of *Brassica* vegetables had various consequences: (i) marketing of I3C as a food supplement (mainly via the internet); (ii) various early-phase clinical trials in the treatment of breast cancer, cervical dysplasia and other diseases (compiled in [29]) and the search for congeners with higher efficacy in the prevention and treatment of cancer [30,31,32,33,34,35]. Specifically, it was found that N-substitution with benzyl or alkoxy residues can lead to drastic increases in growth inhibition and various molecular effects in cancer cells in culture. Generally, these congeners were chemically more stable (due to electron-withdrawing effects of the substituents) and more lipophilic than I3C. Although several different molecular mechanisms appear to be involved in the biological activities of I3C and its derivatives, the following observation is pivotal in the context of our study: Rothman et al. [34] observed that the sensitivity of nearly 500 cancer cell lines toward *N*-(2-pyridylmethyl)-I3C and *N*-(*p*-methoxybenzyl)-I3C was highly correlated (*p* < 10^−18^) with their sulphotransferase (SULT) 1A1 expression. Using another congener, containing a benzyl-derived substituent with a fluorescent marker, they found covalent modification of numerous proteins. A further experimental study and computational analyses indicated that hundreds of candidate drugs (small molecules, including *N*-benzyl-I3Cs) for the treatment of cholangiocarcinoma and hepatocellular carcinoma require bioactivation by SULT1A1 to alkylating metabolites [35].

In the preceding section, we present potentially beneficial effects of I3C and its derivatives. We turn to potentially adverse effects. I3C demonstrated tumour-promoting activities in various animal models involving an initiating treatment with a chemical carcinogen followed by long-term administration of the potential promotor [36,37,38]. NTP conducted a 24-month carcinogenicity study in Harlan Sprague Dawley rats and B6C3F1/N mice with I3C [29]. There was “no evidence of carcinogenic activity” in male rats and female mice. However, there was “some evidence of carcinogenic activity” of I3C in female rats based on increased incidences of malignant uterine neoplasms; additionally, fibroma and fibrosarcoma occurred in the skin. And there was “clear evidence of carcinogenic activity” of I3C in male mice based on increased incidences of liver neoplasms (hepatocellular adenoma, hepatocellular carcinoma, and hepatoblastoma).

A limited number of genotoxicity tests were performed with I3C. It was weakly mutagenic in some experiments with *Salmonella typhimurium* TA100, whereas all other *S. typhimurium* and *Escherichia coli* strains used provided negative or equivocal results [29,39]. Likewise, the result of a gene mutation test in L5178Y *tk^+^*^/*−*^ cells was negative [39]. No induction of chromosomal aberrations was observed in the bone marrow of mice treated orally or intraperitoneally with I3C [40,41]. After gavage of I3C, increased frequencies of micronuclei were observed in polychromatic erythrocytes in the bone marrow in female mice, but not in males [39]. The result was negative in a similar study with rats [29]. No increases in micronuclei were detected in peripheral normochromatic erythrocytes of male and female mice following treatment with I3C by gavage for three months [29].

Feeding of broccoli or cauliflower to rats led to the formation of characteristic DNA adducts in the gastro-intestinal tract, the liver, and various other tissues [42]. The structure of these adducts (Scheme 1) was identified by mass spectrometry and NMR analysis [43]. The adducts were formed from metabolically activated nGBS. They could be generated by incubating DNA with nGBS in the presence of myrosinase, probably via a benzylic cation spontaneously formed from 1-methoxy-3-indolylmethyl isothiocyanate (Scheme 1) [43]. However, the same adducts were also formed, when bacterial or mammalian cells engineered for expression of human SULT1A1 were treated with NI3C [43]. The formation of these adducts led to the induction of gene mutations in bacterial and mammalian cells; another genotoxic effect was the induction of sister chromatid exchange (SCE) in mammalian target cells [44]. nGBS as well as NI3C, when administered by gastric gavage to mice, led to the formation of these DNA adducts [45], and also of protein adducts [46]. However, the tissue distribution of the adducts was very different—adduction by nGBS was focussed on the large intestine, probably due to its activation by bacterial glucosidases. NI3C formed high levels of DNA adducts in tissues with high expression of Sult1a1, i.e., liver, caecum, and colon [45,47]. Knockout of Sult1a1 drastically reduced the adduction in these tissues, by 98, 99, and 89%, respectively [47]. Introduction of the human *SULT1A1-SULT1A2* gene cluster into Sult1a1-knockout mice re-established high adduct formation by NI3C and expanded it to further tissues, in line with the broader expression of human SULT1A1 compared to the mouse Sult1a1 [47]. Interestingly, adduct formation was high and largely unaffected by the SULT1A1/Sult1a1 status in the stomach, implying a different activation mechanism (e.g., by another Sult form or acid-mediated) [47]. Adduct formation was particularly low in the bone marrow in all mouse models (<1% of adduct levels found in liver, caecum, and stomach of wild-type mice) [47]; the finding is important, since various common in vivo genotoxicity tests are conducted in bone marrow cells.

Otteneder and Lutz analysed the relationship between adduct levels and carcinogenicity of chemicals in the liver of mice and rats (adducts after repeated treatments, at least 10 times; tumours in two-year bioassays). In the mouse, they ranged from 812 to 5543 per 10^8^ nt at the TD50 of the chemicals (dose required for tumorigenesis in 50% of the treated animals, corrected for spontaneous tumours) [48]. A single oral dose of NI3C (106 mg per kg body mass) was sufficient to form 5190 DNA adducts per 10^8^ nucleotides (nt) in the liver of wild-type mice (dA and dG adducts, structures in Scheme 1) [47]. This value was increased to 9960 adducts per 10^8^ nt in mice carrying the human *SULT1A1-SULT1A2* genes [47]. In the latter model, adduct data are additionally available for repeated treatments (using a lower individual dose, 26 mg per kg body mass); the resulting adduct levels amounted to 1750, 6060, and 11,470 adducts per 10^8^ nt after 1, 10, and 40 treatments, respectively [49]. With these adduct levels, it can be expected that NI3C would be carcinogenic in mice (wild-type mice as well as mice transgenic for *SULT1A1-SULT1A2*) in two-year bioassays. This view is supported by the results of a genome-wide expression analysis in mouse liver: NI3C treatment up-regulated many mediators of the tumour suppressor p53 and down-regulated *Fhit* and other long genes [49]. Overall, the expression changes following NI3C treatment were similar to the expression signature caused by known genotoxic hepatocarcinogens [49].

The DNA adduct formation in mice by NI3C were remarkably similar to that formed by methyleugenol, regarding adduction site (exocyclic amino group of purine bases), adduct level per dose unit, tissue distribution, and modulation by the genetic SULT1A1/Sult1a1 status [47,50]. Methyleugenol was classified into group 2A (probably carcinogenic to humans) by the International Agency for Research on Cancer [51].

The work on broccoli, nGBS and NI3C, as described in the preceding paragraphs, was conducted in our laboratory and in collaboration with other groups. The research group no longer exists following retirement. Here we report various unpublished findings on the bioactivation and genotoxicity of NI3C and I3C. They might be helpful, among others, in the development of N-substituted I3Cs as potential cancer drugs.

## 2. Results

### 2.1. Mutagenicity in Bacteria

**Figure 1:**
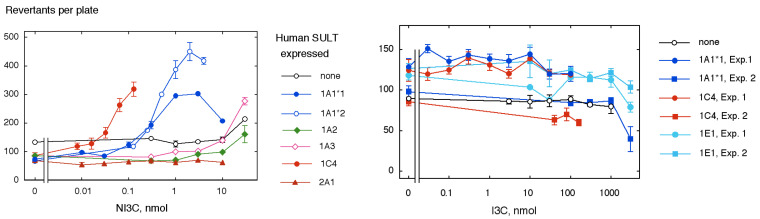
Mutagenicity test of *N*-methoxyindole-3-carbinol (NI3C, **left** panel) and indole-3-carbinol (I3C, **right** panel) in *S. typhimurium* TA100-derived strains engineered for expression of various human sulphotransferase (SULT) forms. Values are means ± SE of 3 plates (treatment groups) or six plates (vehicle controls). Notes: (1) Mutagenicity data for NI3C in strains TA100 and TA100-hSULT1A1*1 have been published before [44]; the data in the figure are from an unpublished repeat experiment (with similar results); data for repeat experiments with SULT1C4 are displayed in Figure S1; (2) Two data sets are presented for I3C tested in SULT-expressing strains.

We previously investigated the mutagenicity of NI3C in the conventional *S. typhimurium* strain TA100 and a TA100-derived strain expressing human SULT1A1 [44]. The expression of SULT1A1 (*1 reference allelic variant) enhanced the mutagenicity of NI3C nearly 200-fold, as based on the initial slopes of the dose–response curves (3 and 600 revertants per nmol). We further investigated NI3C in bacterial strains expressing other human SULT forms (Figure 1, left panel; Figure S1). SULT1A1*2, a common allelic variant [52], activated NI3C similarly to the reference form SULT1A1*1. No activation was observed with SULT1A2 and SULT1A3, the other members of the SULT1A subfamily, as well as SULT2A1, the major form of the SULT2 family. However, activation was detected with SULT1C4, a form primarily expressed in foetal tissues [53,54]. The resulting mutagenicity was even stronger, i.e., detected at lower dose levels, than with SULT1A1 (2900 versus 600 revertants per nmol).

Furthermore, we assessed the mutagenicity of the congener, I3C, in the parental strain TA100 and TA100-derived strains expressing SULT1A1, 1C4, and 1E1 (Figure 1, right panel). All results were negative, although I3C could be tested across much higher dose levels than NI3C due to lower bacteriotoxicity.

### 2.2. Induction of Sister Chromatid Exchange (SCE) in Mammalian Cells in Culture

In the preceding study, NI3C induced the formation of SCE in parental V79 cells (SULT-deficient) and V79-hSULT1A1 cells (engineered for expression of human SULT1A1) [44]. The SULT1A1 reduced the concentration required for a robust increment of SCE (ΔSCE ≥ 3 per metaphase) from 10 to 0.3 µM. After detecting that SULT1C4 is more efficient than SULT1A1 in the activation of NI3C in a bacterial mutagenicity test (Section 2.1), we wanted to verify this finding in mammalian cells. We used a V79-derived cell line co-expressing human CYP1A2 and SULT1C4, as we do not have a cell line only engineered for SULT1C4 expression. NI3C was tested for SCE induction in this cell line across eight concentrations ranging from 0.01 to 30 µM (Table 1). At all these concentrations, ΔSCE per metaphase was ≥3 and statistically highly significant (*p* < 10^−4^); thus, the activation was clearly more efficient than in V79-hSULT1A1 cells.

The SCE findings obtained with V79-hCYP1A2-SULT1C2 cells imply that either CYP1A2 or SULT1C2 (SULT1C4 according to a newer nomenclature), or a combination of both enzymes is capable of converting NI3C into a genotoxic metabolite. At present, no information is available regarding the biotransformation of NI3C in mammalian systems, apart from evidence that certain SULT enzymes can convert it into electrophilic intermediates (Scheme 1). NI3C is a small molecule containing a hydroxyl group (primary alcohol). Such compounds are often excellent substrates for alcohol dehydrogenases and conjugating enzymes, whose catalytic turnover rates may exceed those of CYP-mediated reactions by several orders of magnitude. We therefore strongly favour the interpretation that the activation observed in V79-hCYP1A2-hSULT1C2 cells is mediated by SULT1C2 (SULT1C4) rather than CYP1A2. This interpretation is further supported by the observation of SULT1C4-mediated mutagenicity in *S. typhimurium* (Section 2.1) at comparable substrate concentrations.

The congener, I3C, has not been investigated for SCE induction in V79-derived cells previously. This was investigated in the present study (Table 2). In the first experiment V79p cells (control cells, containing a puromycin resistance marker, used for the construction of SULT-proficient cell lines) and V79-hSULT1A1 cells were concurrently investigated. The result for I3C was positive in both cell lines, but with a large difference in the concentration of I3C required. In V79p cells, an increase in SCE was only observed at the highest concentration used (200 µM; ΔSCE per metaphase: 6.18; *p* > 10^−5^), whereas the SCE frequencies at the lower three concentrations (0.2–20 µM) were similar to those in the negative control. In contrast, V79-hSULT1A1 cells showed increased SCE frequencies across all I3C concentrations used, even at 0.02 µM (with the limitation that the increase at the second lowest concentration was not corroborated statistically). The ΔSCE ≥ 3 threshold was reached at 2 µM I3C in V79-hSULT1A1 cells in this experiment. A repeat experiment, using narrower spacing of the concentrations, was conducted in V79-hSULT1A1 cells; the results confirmed that I3C is able to induce SCE over a very wide concentration range. However, ΔSCE ≥ 3 threshold was only reached at the highest two concentrations of I3C (30 and 100 µM) in this experiment. Nevertheless, ΔSCE of >2.4 (*p* < 0.001) were seen at additional concentrations (0.1, 1, and 3 µM). When concentration–response curves are strongly sublinear (as in this case), it is difficult to define a robust measure for the potency of SCE induction. Saturation of the toxifying enzyme may be a factor determining the concentration–response curve. Other potential mechanisms are discussed in Section 3.1.

### 2.3. Induction of Hprt Gene Mutations in Mammalian Cells in Culture

We previously reported that NI3C induces gene mutations (at the *Hprt* locus) in V79-hSULT1A1 cells [44]. This effect was observed at concentrations of 1.5–3 µM; at the next higher concentration (6 µM) mutagenicity was overshadowed by cytotoxicity (relative cell number reduced to 40% of the vehicle control value). Data with control cells and cell lines expressing other SULT forms were not published at that time. This is now resumed in the present study (Table 3). NI3C was not mutagenic in V79 control cells. Moreover, it only showed low cytotoxicity—survival was high even at 96 µM NI3C; interestingly, acute toxicity remained low at the next higher concentration (192–384 µM), but toxicity was manifested with delay in the second subculture after treatment. NI3C was more cytotoxic in V79-hCYP1A2-SULT1C2 (Table 3) compared to V79-SULT1A1 cells (data in [44]). In the initial experiment in V79-hCYP1A2-SULT1C2 cells, the cell number was reduced even at the lowest NI3C concentration used (0.375 µM), and no significant cell survival occurred at 6 µM. The mutant frequencies were increased statistically significantly at 0.75 and 1.5 µM (*p* < 0.01 and 0.0001, respectively). However, this finding must be taken with the reservations that these mutant frequencies were within historical negative control values and were associated with relatively high cytotoxicity. Therefore, 0.375 µM was used as the highest concentration in the repeat experiment. The cytotoxicity was negligible in this experiment, but no mutagenic effect was detected either.

The congener, I3C, has not been investigated for the induction of gene mutations in V79-derived cells previously. This is now addressed in the present study (Table 4). In the first experiment V79 and V79-hSULT1A1 cells were concurrently exposed to I3C for 2 h. Cytotoxicity was moderate even at the highest I3C concentration used (1600 µM); no mutagenicity was detected at any concentration in either cell line. The exposure time was prolonged to 24–72 h in follow-up experiments. This modification enhanced the cytotoxicity of I3C in both cell lines. No mutagenicity was detected in wild-type V79 cells. However, a slight nominal increase in the mutant frequency was observed at the highest test concentration in all three experiments conducted in V79-hSULT1A1 cells with prolonged exposure time (24–72 h)—for some treatment groups this increase reached statistical significance. We hypothesise that these increases reflect true, but very weak SULT-dependent mutagenicity. However, these findings do not meet our standard criteria for a clear positive test result.

Comparing the cytotoxicity of I3C and NI3C (as detected within the SCE and Hprt gene mutation assays), two statements apply: (1) I3C is clearly less cytotoxic than NI3C in V79 cells as well as V79-hSULT1A1 cells; (2) the expression of SULT1A1 strongly enhanced the cytotoxicity of NI3C, whereas it showed low (or no) effect on the cytotoxicity of I3C.

### 2.4. Cellular Localisation of NI3C DNA Adducts and SULT1A1/2 Enzyme Protein in the Kidneys of FVB/N-hSULT1A1/2 Mice Treated with NI3C

**Figure 2:**
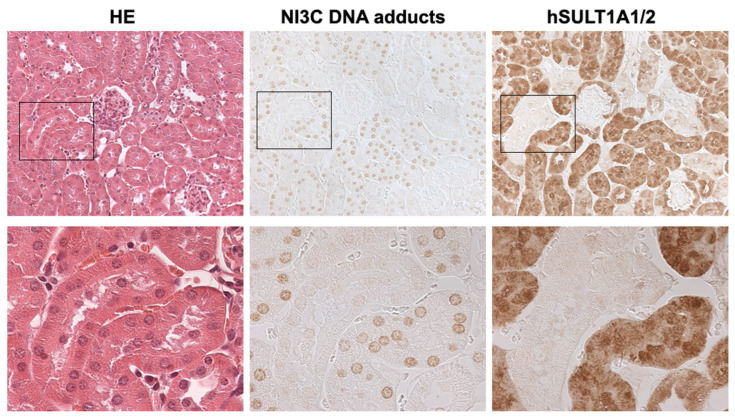
Immunohistochemical localisation of NI3C DNA adducts and human SULT1A1/2 protein in kidneys of male FVB/N-hSULT1A1/2 mice treated with NI3C. Treatment was conducted by gavage, three times per week, 28 mg/kg body mass (in total 40 treatments in 90 d). The figure shows three serial sections of the kidney. One section was stained with haematoxylin/eosin (**left**), the other sections were immune-stained using antisera that specifically recognise NI3C DNA adducts (**middle**) and human SULT1A1/2 protein (**right**). The lower row shows a magnification of the area marked by the rectangle in the upper row.

FVB/N-hSULT1A1/2 mice were sub-chronically treated with NI3C by gavage. Then their kidneys were immunohistochemically analysed for the presence and distribution of NI3C DNA adducts and human SULTlA1/2 protein (Figure 2). The kidneys showed areas of tubular cells with high levels of SULT1A1/2, whereas the glomeruli and other areas of tubular cells remained unstained. Similar findings were made in animals not treated with NI3C (Figure S2 in the Supplementary Materials). The SULT1A1/2 protein was distributed over the entire cell, in the cytoplasm and apparently also in the nuclei. The antibody raised against NI3C DNA adducts only stained nuclei. Most cells with high SULT1A1/2 expression had nuclei showing clear positive reaction with the antibodies raised against the DNA adducts. However no adduct-positive nuclei were observed in kidney areas with low (or undetectable) SULT1A1/2 protein expression. The finding suggests that the reactive metabolites formed from NI3C are not transferred between cells, but only act in the cells in which they are generated.

## 3. Discussion

### 3.1. Technical Aspect: Concentration–Response Curves in the SCE Test

The SCE assay using the fluorescence-plus-Giemsa technique was introduced by Latt in 1974 [57] and remained one of the most widely used genotoxicity assays for approximately 25 years [58,59]. However, since SCE events themselves may not directly result in toxicological consequences, the assay indicates genetic damage only indirectly. Consequently, the test was gradually replaced by newer assays with more biologically relevant or mechanistically interpretable endpoints.

Nevertheless, we still consider the SCE assay highly valuable for studies investigating the bioactivation of chemicals in engineered cell lines expressing xenobiotic-metabolising enzymes. One important practical advantage is that only small numbers of cells and, therefore, limited quantities of test compounds are required. This is particularly relevant because many compounds used in our studies must either be purified from natural products or synthesised chemically, as they are not commercially available.

Historically, the SCE assay was mainly used to classify compounds as either positive or negative. Typically, they were tested in a relatively narrow concentration range close to cytotoxic levels; concentration–response relationships were of minor interest. In contrast, our primary objective is to determine which enzyme isoforms are capable of activating a given compound. Since activation at low substrate concentrations is especially relevant for dietary and environmental mutagens, we routinely investigate broad concentration ranges.

Interestingly, several compounds have produced positive results across remarkably broad concentration ranges. One example is NI3C in V79-hCYP1A2-hSULT1C2 cells (Table 1). Clear positive effects (ΔSCE per metaphase ≥ 3 and *p* < 10^−4^) were observed at concentrations ranging from 0.01 to 30 µM NI3C. However, despite a 3000-fold increase in concentration, the effect size increased by only a factor of 3.3, indicating a strongly hypolinear concentration–response relationship.

Several mechanisms may explain this phenomenon, including saturation of the activating enzyme, substrate inhibition (strong for various SULT forms and substrates [60,61]), depletion of the cofactor PAPS (present in cells at low levels [62,63]), and induction of recombination-associated proteins following DNA damage. Regardless of the precise mechanism, the flat concentration–response relationship complicates the definition of a robust quantitative potency parameter.

Previously, we used the concentration required to achieve ΔSCE per metaphase ≥ 3 as an indicator of activation efficiency. In the present study, however, two independent experiments examining I3C in V79-hSULT1A1 cells yielded substantially different threshold concentrations: 2 µM in experiment 1 and 30 µM in experiment 2. If statistical significance had instead been used as the criterion, the corresponding concentrations would have been more similar (0.02 and 0.1 µM, respectively). Importantly, all of these concentrations were markedly lower than those required to induce effects in control V79p cells (200 µM). Therefore, we have no doubt that I3C undergoes bioactivation mediated by SULT1A1.

### 3.2. NI3C

It has previously been shown that NI3C can be metabolised to a reactive genotoxic intermediate by human SULT1A1 (see Introduction). Here we demonstrate that human SULT1C4 is even more efficient in this activation, based on the result obtained with recombinant bacteria (induction of gene mutations) and mammalian cells (cytotoxicity and induction of SCE). The induction of gene mutations in SULT1A1-expressing V79 cells was observed in a narrow concentration window limited by cytotoxicity. This effect was borderline positive in one experiment and absent in the repeat experiment in SULT1C4-expressing V79 cells. Possibly, different kinetics (faster/slower activation) altered the balance between mutagenicity and genotoxicity.

SULT1A1 is the most abundant SULT in the human organism [64,65]. The protein was detected in many tissues, its levels being particularly high in liver as well as small and large intestine. On the contrary, information on the expression of SULT1C4 is largely limited to the mRNA level. High expression was observed in foetal liver, kidney, and lung [53,54]. In the adult organism, expression was detected in most tissues studied, with low tissue specificity [53,54]; it was highest in ovary (and other female reproductive tissues), gallbladder, and brain [54,66]. To the best of our knowledge, SULT1C4 protein was only detected in liver of early foetuses [53] and Caco-2 cells (derived from a colorectal adenocarcinoma) [67].

Treatment of wild-type mice with NI3C led to the formation of high levels of DNA adducts in four tissues: liver, stomach, caecum, and colon. Knockout of Sult1a1 largely abolished the adduction in liver and large intestine, whereas the decrease in the adducts was moderate (<50%) in the stomach, in agreement with the low gastric Sult1a1 expression [68]. The finding implies the occurrence of an additional activation mechanism in the stomach. One possibility is acid-mediated formation of the benzylic cation, eventually followed by chlorination (exchange of the leaving group) [69]. Another possibility is activation by a different Sult form. Indeed, the expression of Sult1c2 is higher in the stomach than in any other tissue investigated, and several other Sult forms (1b1, 1c1, 2b1, 1d1, and 5a1) are expressed in this tissue at appreciable levels [68].

NI3C led to the formation of high levels of DNA adducts in the kidney of mice expressing transgenic human SULT1A1/2. This finding raised the question on the site of activation, extra-renally followed by transfer via the blood circulation and uptake into kidney cells, as observed with 1-sulphooxymethylpyrene and 5-sulphooxymethylfurfural [50], or local activation. The findings reported in Section 2.4—high adduct levels only in cells with high SULT1A1/2 expression—argue for local activation without significant transfer of the active metabolite to neighbouring cells.

A reviewer asked whether the NI3C concentrations required to induce positive results in the in vitro genotoxicity assays could realistically be achieved in human tissues following dietary exposure. Indeed, NI3C produced a robust positive response in the SCE assay using V79-hCYP1A2-hSULT1C2 cells at concentrations as low as 10 nmol/L (Table 1). Similarly, a concentration of approximately 20 nmol/L was sufficient to produce a 1.5-fold increase in revertants in *S. typhimurium* TA100-hSULT1C2 (calculated from Figure S2).

nGBS is currently the only known dietary precursor of NI3C. Steinbrecher and Linseisen estimated the average daily intake of nGBS in the EPIC-Heidelberg cohort to be 0.68 mg in males and 0.60 mg in females [70], corresponding to 21 and 19 nmol/kg body mass, respectively, for a 70 kg individual. However, dietary exposure may vary substantially. For example, a typical level of nGBS in broccoli is 7.91 mg per 100 mg fresh mass [70]. Consumption of 200 g broccoli would therefore correspond to an intake of around 472 nmol/kg body mass in a 70 kg individual.

These calculations demonstrate that dietary intake of nGBS can be relatively high compared with the NI3C concentrations required to induce genotoxic effects in vitro. However, toxicokinetic considerations in vivo are far more complex. Most importantly, biological effects are likely to depend not simply on the concentration of NI3C itself, but rather on the extent of conversion to reactive intermediates at relevant target tissues. DNA and protein adducts detected in cellular and animal models (Scheme 1) may therefore represent useful biomarkers for estimating exposure in human tissues and blood samples.

### 3.3. I3C

The enzymatic hydrolysis of many glucosinolates leads to the formation of isothiocyanates that are sufficiently stable for their identification [6,7]. The situation is different for the indole glucosinolates, with carbinols rather than isothiocyanates as relatively stable breakdown product. However, Hanley et al. detected a short-lived intermediate in the enzymatic hydrolysis of nGBS and identified it as the corresponding isothiocyanate (structure in Scheme 1) based on its molecular mass and fragmentation pattern [11,12]. Such an intermediate could not be detected with GBS—probably it was too short-lived due to the lack of the stabilisation by the electron-withdrawing methoxy group [11,12]. However, incubation of GBS with herring sperm DNA in the presence of myrosinase resulted in the formation of high levels of DNA adducts, similar to the levels obtained with nGBS [71], implying the presence of an electrophilic intermediate, probably the isothiocyanate. When the herring sperm DNA was replaced by intact bacteria (*S. typhimurium* TA100), the concentration of GBS had to be increased nearly tenfold above that of nGBS for generating comparable levels of DNA adducts [71]. Probably, the reactive intermediates formed from GBS were much less efficient in penetrating the bacteria that those formed from nGBS—putatively due to higher chemical reactivity.

Sulphuric acid (for both protons) is a much stronger acid than isothiocyanic acid. Therefore, the sulphates of I3Cs may be even more reactive and short-lived than the corresponding isothiocyanates, rendering their detection in standard enzyme activity assays futile. In the present study, we observed that I3C strongly induces SCE in V79-hSULT1A1 cells, while this activity was very weak in parental V79 cells. This finding implies that I3C is metabolised by SULT1A1 to a reactive sulpho conjugate. However, SULT1A1 had no effect (or only minor effects) on the mutagenicity and cytotoxicity in bacterial and mammalian target cells. This differential response of the toxicological endpoints is not yet understood—possibly the highly reactive intermediates (benzylic sulphate and/or cation) only reach certain target sites in the cells and in the chromosomes; or the pattern of adducts (positions in the target molecules) may be affected.

It is not known whether the observed SULT-mediated bioactivation is involved in the carcinogenicity observed in the NTP study [29], as to date, no toxicological studies have been conducted with I3C in mouse models with modified SULT status.

I3C did not show any mutagenicity in our experiments with *S. typhimurium* strain TA100 or any TA100-derived strains expressing human SULTs (including 1A1 and 1C4) (Section 2.1). This finding stands in some contrast to two previous studies, reporting “weakly positive” results in TA100 in the absence and presence of an external metabolising system [29,39]. However, three experiments were conducted in one of these studies. In the direct test, one experiment gave the “weakly positive” result, whereas the remaining experiments gave “negative” results [29]. Such variation might be attributed to the limited chemical stability of I3C, as illustrated by its condensation under acidic conditions. Moreover, we used somewhat different exposure conditions compared to the other studies, e.g., less DMSO per incubation (10 versus 50 µL), longer liquid preincubation (60 versus 20 min), higher bacterial density and another exposure medium (100 mM MgSO_4_, optimised for the synthesis of PAPS, the co-substrate for SULTs).

### 3.4. N-Substituted I3Cs Other than NI3C

Various N-substituted I3Cs demonstrated strong antiproliferative activity in human cancer cells in culture [30,31,32,33,34,35]. This activity was dependent on SULT1A1-mediated bioactivation. Covalent binding to proteins was observed with a congener carrying a fluorescent substituent at the indole nitrogen [34]. No DNA binding was detected in this study—with the limitation that the assay was not validated and no limit of detection was given. Moreover, what may be true for this congener with a large substituent, may not reflect the situation with other congeners, as their reactive sulfo-conjugates will differ in reactivity, lipophilicity, and steric parameters. The example of NI3C demonstrates that members of this chemical class may extensively bind to DNA after SULT-mediated bioactivation, in addition to binding to proteins. DNA modification may be important in anti-proliferative (anti-neoplastic) activities as well as undesired side-effects. Therefore, it is important that the extent and pattern of genotoxic effects are elucidated. The comparison of I3C and NI3C shows that large differences may occur in this respect within this chemical class.

Hitherto, bioactivation of I3Cs was primarily described for the enzyme SULT1A1. However, in the present study we demonstrate that at least one congener, NI3C, is more efficiently activated by SULT1C4 than by SULT1A1. Whether other N-substituted I3Cs are activated by forms other than SULT1A1 is unknown, but should be clarified. Regarding SULT1C4, it is difficult to assess a possible pharmaco-toxicological significance, as it is a very minor SULT form in the adult human, in contrast to SULT1A1, which is present at moderate to high levels in numerous tissues. However, it cannot be excluded that individual cell types highly express SULT1C4 protein.

Oral administration of NI3C to mice led to the formation of high levels of DNA adducts in stomach, large bowel, and liver [45,47]. In genetically modified mice carrying the human *SULT1A1-SULT1A2* gene cluster, small intestine was a further major adduction site. Knockout of the endogenous Sult1a1 (in the absence of the human SULT1A1/2 transgene) largely abolished the adduction in liver and gut, but not in stomach [47]. We would expect that the oral administration of other N-substituted I3Cs (candidates for treatment of liver tumours [35]) would also lead to metabolism and formation of macromolecular adducts at these sites—stomach, large bowel, and liver. Administration in acid-resistant capsules may reduce the adduction in the stomach, but not in the gut. The latter might lead to undesired side-effects in the gut and reduced hepatic and systemic bioavailability. Parenteral treatment routes may be an alternative.

NI3C forms DNA adducts within the cells mediating its bioactivation, without significant transfer of the active metabolites (benzylic sulphate and cation) into neighbouring cells (Section 2.4) or via the circulation [47], probably owed to their charge and short lifespan. Transfer of reactive metabolites formed from I3C (and GBS) appears to be even more restricted due to their high reactivity. Limitations in the transfer are also expected for reactive metabolites generated from other N-substituted I3Cs. This effect might be helpful in the targeting of cancer cells.

In conclusion, the usage of SULT-dependent alkylators as pharmaceutical drugs provides special chances and challenges. Findings with I3C and NI3C may be used as guide in further investigations.

## 4. Materials and Methods

### 4.1. Chemicals

*N*-Methoxyindole-3-carbinol (NI3C), also named 1-methoxy-3-indolylmethyl alcohol (1-MIM-OH) in some publications, was prepared in the laboratory of Albrecht Seidel (Biochemical Institute for Environmental Carcinogens, Prof. Dr. Gernot Grimmer-Foundation, Grosshansdorf, Germany), as described previously [44]. Indole-3-carbinol (I3C) was purchased from Sigma–Aldrich (Taufkirchen, Germany).

### 4.2. Experimental Models Engineered for Expression of Human SULT Enzymes

Human SULTs (reference type sequences, unless specified otherwise) were expressed in *S. typhimurium* TA100 using the pKKneo vector, as described previously [72,73]. To be consistent with our previous publications, we stayed with the original designation of SULTs in the names of the strains. This is relevant for SULT1C1 and SULT1C2 [55], which have been renamed to SULT1C2 and SULT1C4 by Blanchard et al. [56]. The SULT protein levels in the recombinant strains are given in [73]; the expression of SULT1A1 in TA100-SULT1A1 is in the upper range of that observed in human liver samples [74]. All SULT-expressing strains used in the present study are able to activate 1-hydroxymethylpyrene to a mutagen, which was used as a positive control compound in this mutagenicity test.

Chinese hamster V79 cells (subline V79-MZ) were used as recipient organisms for the vectors encoding human SULTs and CYPs. Various enzymatic properties of that cell line have been described [75]. The control cell line, V79p, was only transformed with the puromycin resistance marker [76]. V79-hSULT1A1 cells (previously named V79-hP-PST) stably express human SULT1A1, at a level in the upper range of that observed in human liver samples [74]. 2-Nitropropane was used as a SULT-dependent positive control compound with this cell line. The cell line V79-hCYP2A1-hSULT1C2 was constructed by transfecting an expression vector encoding human SULT1C2 (old nomenclature [55], or SULT1C4 in the nomenclature by Blanchard et al. [56]) into a V79-MZ-derived cell line expressing human CYP1A2 [77]. Expression of SULT was verified by the demonstration of sulphotransferase activity with the substrate *p*-nitrophenol and mutagenicity of 1-hydroxymethylpyrene.

The construction of FVB/N-SULT1A1/2 mice was described previously [78]. For toxicological studies, hemizygous males of the mouse line originally named tg1 were used. These animals contain multiple copies of the human *SULT1A1-SULT1A2* gene cluster integrated into chromosome 9. The hepatic level of SULT1A1 protein in hemizygous young adult male mice (as used in the toxicological studies) is nearly eight-fold higher than the highest values observed in human liver samples [78].

### 4.3. General Comment on All In Vitro Tests

All genotoxicity tests with bacteria and V79-derived cells were conducted as in our previous study with NI3C [44]. V79-derived cells were maintained in Dulbecco’s modified minimum essential medium supplemented with foetal bovine serum (5%), penicillin (100 units/mL), and streptomycin (100 μg/mL). The cells were grown at 37 °C in a humidified atmosphere containing 5% CO_2_.

### 4.4. Mutagenicity Assay in Salmonella typhimurium Strains

Bacteria were grown and their cell density was adjusted to 5–10 × 10^9^ colony-forming units/mL, as described elsewhere [72]. The bacterial suspension (100 µL) and the test compound (in 10 µL DMSO) were added sequentially to a glass tube containing 500 µL of 100 mM MgSO_4_. After incubation for 60 min at 37 °C, 2 mL warm soft agar (45 °C) (comprising 5.5 mg/mL agar, 5.5 mg/mL NaCl, 50 µM biotin, 50 µM histidine, 25 mM sodium phosphate buffer, pH 7.4) was added, and the mixture was poured onto a Petri dish containing 24 mL minimal agar (15 mg/mL agar in Vogel–Bonner E medium with 20 mg/mL glucose). After incubation for 2–3 days in the dark, the colonies (his^+^ revertants) were counted. Benzo[*a*]pyrene 4,5-oxide and 1-hydroxymethylpyrene were used as directly acting and SULT-dependent positive control compounds, respectively.

### 4.5. SCE Test in V79-Derived Cell Lines

The protocol for the SCE test is described in [79] in detail. Briefly, cells were seeded in 6 cm Petri dishes. After 19 h, the test compound (10 µL DMSO, or the solvent only) and 5-bromo-2′-deoxyuridine were added. Two separate cultures were used for each treatment. After a further 32 h period, colcemid was added. Four hours later, the cells were harvested, treated with hypotonic KCl solution, fixed, stored at 4 °C overnight, and dropped on ice-cold glass slides. The slides were air-dried and stained according to the fluorescence-plus-Giemsa technique. All slides were coded for the evaluation. A total of 25 metaphases containing 20–23 chromosomes were scored for SCE per culture. The number of SCE per metaphase was standardised to 22 chromosomes. The results for both parallel plates were pooled. The frequency of SCE in treatment groups was compared with that observed in the solvent controls using the Mann–Whitney U-test. Concurrently, the proliferation index (proportion of cells in first division + 2 × proportion of cells in second division + 3 × proportion of cells in higher than second division) was determined and used as a measure for cell cycle delay.

### 4.6. Hprt Gene Mutation Test in V79-Derived Cell Lines

Acquisition of resistance towards 6-thioguanine (involving inactivation of the X-chromosomal *Hprt* gene) was used to study the induction of gene mutations in V79-derived cell lines. The experimental protocol was similar to that used in previous studies [44,74,80]. Briefly, 1.5 × 10^6^ cells were added in 30 mL medium on a 15 cm Petri dish (day 0, “initial cultures”). After 18 h, the test compound (dissolved in 60 µL DMSO), or the solvent alone, was added. The exposure was terminated by a change of the medium. On day 4 of the experiment, the cells were detached by treatment with trypsin. Their number, expressed as percent of the corresponding value of the solvent control cultures, was used as a measure of the cytotoxicity of the treatment. The cells were subcultured in normal medium for 3 days, and then (on day 7) subcultured again using 6-thioguanine (7 µg/mL)-supplemented medium for the selection of mutants (10^6^ cells per 15 cm Petri dish; six dishes) and normal medium for determining the total number of colony-forming cells (100 cells per 6 cm Petri dish; three dishes). After 12 additional days, the cultures were fixed and stained with Giemsa; the colonies were counted and the mutant frequencies (MF) calculated for each initial culture. The spontaneous MF (vehicle controls) are <10 per 10^6^ cells in most experiments. In routine tests involving a single experiment and two initial cultures per condition, the test result is considered positive if ΔMF (increase in MF above that of the vehicle control) is ≥40 per 10^6^ cells at one or more concentrations of the test compound, with a plausible concentration–response curve. The test result is considered negative if ΔMF is <10 per 10^6^ cells at all concentrations of the test compound. In all other cases, the result is considered equivocal, and repeat experiment may be required. In extended tests (involving repeat experiments, and/or >2 initial cultures per condition, the test result is considered positive if ΔMF is ≥10 per 10^6^ cells at one or more concentrations of the test compound, and statistically verified.

### 4.7. Animal Experiment

The animal experiment was performed with permission (LUGV V3-2347-28-2011) of the Landesamt für Umwelt, Gesundheit und Verbraucherschutz of the State of Brandenburg, Germany, as described previously [49]. Briefly, the animals were maintained under specific pathogen-free, temperature- and light- (12 h/12 h) controlled conditions. Male hemizygous FVB/N-SULT1A1/2 mice received varying numbers (1–40) of oral treatments with NI3C [150 µmol (26 mg) per kg body mass per treatment], three times per week (always Monday, Wednesday, and Friday), starting at the age of five weeks. Here we report on a small part of this study, using some animals killed after 40 treatments (over a period 90 d).

### 4.8. Immunohistochemistry

Antibodies against NI3C-adducted DNA [45] and human SULT1A1 [65], produced in *E. coli* BL21, were raised in rabbits, as described previously. Tissue was fixed in 4% neutral buffered formalin for 24 h, flushed under tap water for 24 h, dehydrated, and embedded in paraffin wax. Serial sections (2 μm) were processed for histopathology (haematoxylin and eosin staining) and immunohistochemistry. For immunostaining, heat-mediated antigen retrieval was performed in a microwave with target retrieval solution (citrate pH 6). Primary antibodies were diluted in antibody diluent (NI3C adducts: 1:6000; human SULT1A1: 1:1500) and applied overnight at 4 °C. Antibody binding was visualised by N-Histofine^®^ Simple Stain™ Mouse MAX PO Anti-Rabbit and diaminobenzidine according to manufacturer’s instructions. (Nichirei Biosciences, Tokyo, Japan), and 3,3-diaminobenzidine according to manufacturer’s instructions.

Both antisera used are highly specific. This was documented for the antibodies against NI3C DNA adducts in a previous study [45]. As shown in Figure S2 in the Supplementary Materials, anti-human SULT1A1 produced strong staining of some cells in the kidney of FVB/N-SULT1A1/2 mice, whereas no staining occurred in wild-type FVB/N mice.

## 5. Conclusions

I3C and NI3C are genotoxic in the presence of an appropriate SULT, but with large quantitative and qualitative differences: I3C required higher concentrations and induced only SCE, virtually no gene mutations. Other *N*-substituted I3C derivatives, candidate antineoplastic drugs, strongly inhibited the growth of human cancer cells via SULT1A1-mediated formation of electrophilic intermediates. They have not yet been investigated for genotoxicity. We suspect that they may greatly vary in this activity, which in turn might affect their clinical effectiveness as well as adverse side-effects. Likewise, some of these candidate drugs may not only be activated by SULT1A1, but also by other human SULT forms (e.g., SULT1C4).

## Figures and Tables

**Table 1:** *N*-Methoxyindole-3-carbinol (NI3C) tested for induction of sister-chromatid exchanges (SCE) in V79-hCYP1A2-SULT1C2 cells ^1^.

Test Compound	Concentration, µM	Proliferation Index ^2^	SCE per Metaphase ^3^
	0	2.13 ± 0.04	8.91 ± 0.43
NI3C	0.01	2.11 ± 0.00	*12.34 ± 0.59 ^d^*
	0.03	2.10 ± 0.01	*13.55 ± 0.78 ^e^*
	0.1	2.17 ± 0.04	*13.82 ± 0.68 ^e^*
	0.3	2.18 ± 0.05	*18.99 ± 1.01 ^e^*
	1	2.09 ± 0.01	*19.57 ± 1.02 ^e^*
	3	2.14 ± 0.01	*19.69 ± 0.88 ^e^*
	10	2.10 ± 0.00	*19.56 ± 0.77 ^e^*
	30	1.90 ± 0.08	*20.33 ± 0.78 ^e^*
1-HMP ^4^	0.3	1.98 ± 0.02	*29.17 ± 1.21 ^e^*
	3	1.95 ± 0.00	*32.41 ± 1.37 ^e^*

^1^ NI3C was previously tested under equal experimental conditions for SCE induction in V79 and V79-hSULT1A1 cells. The concentration of SCE required for a robust increase in the number of SCE per metaphase (≥3) was 10 and 0.3 µM, respectively. V79-hCYP1A2-hSULT1C2 cells are engineered for expression of human CYP1A2 and SULT1C2 (according to the original nomenclature [55]; corresponding to SULT1C4 of the actual nomenclature [56]). ^2^ Mean ± half range of 2 cultures. ^3^ Mean ± SE of 50 metaphases (25 each from two cultures). Superscript letters indicate statistically significant differences from the negative control using the Mann–Whitney U-test: no letter, *p* ≥ 0.05; (d) 10^−4^ > *p* ≥ 10^−5^; (e) *p* < 10^−5^. Italics indicate values exceeding the corresponding solvent control value by at least 3 SCE per metaphase (as in the preceding study with NI3C [44]). ^4^ 1-Hydroxymethylpyrene, positive control.

**Table 2:** Indole-3-carbinol (I3C) tested for induction of sister-chromatid exchanges (SCE) in V79p and V79-hSULT1A1 cells ^1^.

Cell Line	I3C, µM	Proliferation Index ^2^	SCE per Metaphase ^3^
V79p	0	2.05 ± 0.04	7.96 ± 0.45
	0.2	2.07 ± 0.02	7.58 ± 0.35
	2	2.04 ± 0.01	8.48 ± 0.47
	20	2.08 ± 0.01	7.92 ± 0.35
	200	1.93 ± 0.03	*14.14 ± 0.62 ^e^*
V79-hSULT1A1, experiment 1	0	2.03 ± 0.00	6.86 ± 0.31
	0.02	2.09 ± 0.00	8.86 ± 0.42 ^c^
	0.2	2.09 ± 0.00	7.66 ± 0.38
	2	2.05 ± 0.00	*12.09 ± 0.67 ^e^*
	20	2.11 ± 0.04	*10.82 ± 0.49 ^e^*
	200	1.94 ± 0.01	*20.29 ± 0.77 ^e^*
V79-hSULT1A1, experiment 2 ^4^	0	2.39 ± 0.22	6.99 ± 0.33
	0.03	2.34 ± 0.10	7.33 ± 0.37
	0.1	2.45 ± 0.04	9.40 ± 0.37 ^d^
	0.3	2.31 ± 0.01	7.92 ± 0.37 ^a^
	1	2.33 ± 0.03	9.40 ± 0.36 ^e^
	3	2.24 ± 0.01	9.43 ± 0.48 ^c^
	10	2.30 ± 0.03	8.45 ± 0.36 ^b^
	30	2.36 ± 0.08	*12.59 ± 0.41 ^e^*
	100	2.23 ± 0.00	*19.04 ± 0.81 ^e^*

^1^ V79-hSULT1A1 cells are engineered for expression of human SULT1A1 (at the protein level similar to human liver); V79p is a control line engineered only for the puromycin resistance marker. ^2^ Mean ± half range of 2 cultures. ^3^ Mean ± SE of 50 metaphases (25 each from 2 cultures). Superscript letters indicate statistically significant differences from the negative control using the Mann–Whitney U-test: no letter, *p* ≥ 0.05; (a) 0.05 > *p* ≥ 10^−2^; (b) 10^−2^ > *p* ≥ 10^−3^; (c) 10^−3^ > *p* ≥ 10^−4^; (d) 10^−4^ > *p* ≥ 10^−5^; (e) *p* < 10^−5^. Italics indicate values exceeding the value of the corresponding solvent control by at least 3 SCE per metaphase (as in the preceding study with NI3C [44]). ^4^ Cells treated with the positive control compound, 1-hydroxymethylpyrene (0.3 and 1 µM) showed 20.37 ± 0.67 and 28.19 ± 1.21 SCE per metaphase (*p* < 10^−5^) and proliferation indices of 2.07 ± 0.00 and 2.08 ± 0.08, respectively. No concurrent positive controls were used in experiment 1 (conducted with two cell lines), as they would have exceeded the technical size limit of the experiment. However, extensive historical data are available (see Supplementary Materials, Section S4).

**Table 3:** *N*-Methoxyindole-3-carbinol (NI3C) tested for cytotoxicity and induction of Hprt gene mutations in V79 and V79-hCYP1A2-SULT1C2 cells ^1^.

Cell Line	NI3C, µM	Cell Number, % of Control	Mutants/10^6^ Cells, Mean ± SE of n Cultures ^2^
V79	0	100	5.9 ± 2.1 (3)
	3	109	2.9 ± 1.0 (3)
	6	69	6.5 ± 2.3 (3)
	12	60	6.5 ± 2.3 (3)
	24	117	9.6 ± 5.1 (3)
	48	75	7.4 ± 3.5 (3)
	96	83	9.2 ± 4.5 (3)
	192	73	tox ^3^
	384	35	tox ^3^
	768	<2	tox
V79-hCYP1A2- hSULT1C2, experiment 1	0	100	2.3 ± 0.4 (8)
	0.375	42	2.3 ± 0.5 (4)
	0.75	24	5.3 ± 0.8 (4) ^b^
	1.5	17	7.4 ± 1.1 (4) ^d^
	3	15	0.4 ± 0.4 (4) ^a^
	6	<2	tox
V79-hCYP1A2- hSULT1C2, experiment 2	0	100	2.5 ± 0.7 (8)
	0.047	88	1.5 ± 0.4 (4)
	0.094	104	1.3 ± 0.2 (4)
	0.188	89	1.6 ± 0.3 (4)
	0.375	87	2.9 ± 0.2 (4)

^1^ We previously investigated NI3C in a Hprt gene mutation test with V79-hSULT1A1 cells [44]. It was positive at concentrations of 1.5–3 µM; at the next higher concentrations (6 µM), mutagenicity was masked by cytotoxicity (relative cell number reduced to 40% of the vehicle control value). Now, we examine NI3C in two further cell lines, V79 and V79-hCYP1A2-hSULT1C2. The latter line is engineered for expression of human CYP1A2 and SULT1C2 (according to the original nomenclature; corresponding to SULT1C4 of the actual nomenclature). The exposure time (72 h) and all other experimental conditions were the same, as previously used with V79-hSULT1A1 cells. ^2^ Mean ± SE of n cultures subjected to the corresponding treatment. tox, toxic (number of cells at first or second passage after treatment insufficient for continuation of the experiment). Superscript letters indicate statistically significant differences from the negative control using the two-sided Student’s *t*-test: no letter, *p* ≥ 0.05; (a) 0.05 > *p* ≥ 10^−2^; (b) 10^−2^ > *p* ≥ 10^−3^; (d) 10^−4^ > *p* ≥ 10^−5^. The positive control compounds (±)-*anti*-chrysene-1,2-dihydrodiol-3,4-oxide (5 µM) strongly elevated the mutant frequency in both cell lines. The SULT-dependent mutagen, 1-hydroxymethylpyrene (1.25 µM) was positive in V79-hCYP1A2-hSULT1C2 cells, but not in wild-type V79 cells. ^3^ Although appropriate numbers of cells were harvested at the first subculturing during the expression period, this number was very low at the next cell passage, insufficient for the determination of mutant frequencies. This finding suggests delayed cytotoxicity.

**Table 4:** I3C tested for induction of Hprt gene mutations in V79 and V79-hSULT1A1 cells ^1^.

Cell Line	Exp. Number, Duration of Exposure	I3C, µM	Cell Number, % of Control	Mutants/10^6^ Cells, Mean ± SE of (n) Cultures ^2^
V79	Exp. 1, 2 h	0	100	1.6 ± 0.8 (3)
		200	90	3.0 ± 1.2 (3)
		400	101	3.5 ± 0.7 (3)
		800	96	2.2 ± 0.7 (3)
		1600	76	5.9 ± 5.9 (3)
	Exp. 2 24 h	0	100	0.8 ± 0.0 (3)
		200	91	1.3 ± 0.5 (3)
		400	66	2.1 ± 0.5 (3)
		600	<2	tox
V79-hSULT1A1	Exp. 1, 2 h	0	100	2.8 ± 0.6 (3)
		200	93	2.3 ± 0.1 (3)
		400	124	3.7 ± 0.9 (3)
		800	97	4.6 ± 0.8 (3)
		1600	58	4.4 ± 0.7 (3)
	Exp. 2 24 h	0	100	7.3 ± 0.1 (3)
		10	103	13.9 ± 5.1 (2)
		30	104	9.0 ± 1.5 (2)
		100	86	13.1 ± 2.6 (2)
		300	90	21.4 ± 5.1 (2) ^b^
		1000	<2	tox
		3000	<2	tox
	Exp. 3 24 h	0	100	5.1 ± 0.6 (4)
		50	86	6.5 ± 0.4 (4)
		100	88	9.2 ± 1.1 (4) ^a^
		200	78	9.8 ± 2.0 (4)
		400	43	9.1 ± 1.8 (4)
		800	<2	tox
	Exp. 4 72 h	0	100	2.7 ± 1.4 (3)
		100	95	3.5 ± 0.4 (3)
		200	57	8.6 ± 0.9 (3) ^a^
		400	<2	tox

^1^ Cultures of V79 and V79-hSULT1A1 cells were exposed to the test compounds for 2–72 h. They were subcultured 3 d after the beginning of the exposure period. The number of cells harvested at that time was used as a measure of cytotoxicity of the treatment (column 4). After an additional 4 d period, cells were passaged again and subjected to selection by 6-thioguanine (and determination of cloning efficiency in normal medium). ^2^ Mean ± SE of n cultures subjected to the corresponding treatment. tox, toxic (number of cells at first or second passage after treatment insufficient for continuation of the experiment). Superscript letters indicate statistically significant differences from the negative control using the two-sided Student’s *t*-test: no letter, *p* ≥ 0.05; (a) 0.05 > *p* ≥ 10^−2^; (b) 10^−2^ > *p* ≥ 10^−3^; The positive control compounds (±)-*anti*-chrysene-1,2-dihydrodiol-3,4-oxide (5 µM) strongly elevated the mutant frequency in both cell lines. 2-Nitropropane (5 mM) showed this effect in V79-hSULT1A1 cells selectively.

## Data Availability

The original contributions presented in this study are included in the article. Further inquiries can be directed to the corresponding author.

## Supplementary Materials

The following supporting information can be downloaded at: https://www.mdpi.com/article/10.3390/ph19060895/s1, Figure S1: Mutagenicity of NI3C in *S. typhimurium* TA100-hSULT1C2; Figure S2: Specific detection of human SULT1A1/2 in the kidney of FVB/N-SULT1A1/2 by antibodies raised against human SULT1A1; Table S1: SCE frequency of the negative controls of cell lines V79-MZ and V79-hSULT1A1 (historical controls); Table S2: Compounds that increased the SCE frequency in V79-MZ and V79-hSULT1A1 strongly in our experiments (useful as positive controls). Reference [81] is cited in the Supplementary Materials.

## Abbreviations

The following abbreviations are used in this manuscript:

AhR	arylhydrocarbon receptor
CYP	cytochrome P450
ΔSCE	increase in the number of SCE (per metaphase) in the treatment group compared to the negative control
DMSO	dimethylsulphoxide
GBS	glucobrassicin (3-indolylmethyl glucosinolate)
Hprt	hypoxanthine phosphoribosyltransferase
I3C	indole-3-carbinol
MF	mutant frequency
nGBS	neoglucobrassicin (*N*-methoxy-3-indolylmethyl glucosinolate)
NI3C	*N*-methoxyindole-3-carbinol (1-methoxy-3-indolylmethyl alcohol)
NMR	nuclear magnetic resonance
nt	nucleotide(s)
NTP	National Toxicology Program
PAPS	3′-phosphoadenosine-5′-phosphosulphate (cofactor for SULT)
SCE	sister chromatid exchange(s)
SULT	sulphotransferase (human and generic)
Sult	sulphotransferase (mouse)
