# Supplementary material for: Genotoxicity Studies of Indole-3-carbinol and N-Methoxyindole-3-carbinol—The Effect of Sulphotransferases
Source: Pharmaceuticals (Basel). 2026 Jun 5;19(6):895. doi: 10.3390/ph19060895 (PMC13304936; doi:10.3390/ph19060895)
Supplement: Supplementary file 1 [file pharmaceuticals-19-00895-s001.zip › pharmaceuticals-4265834-supplementary.pdf]

Genotoxicity Studies of Indole-3-carbinol and *N*-Methoxyindole-3-carbinol – the Effect of Sulphotransferases

Supplementary Material

Hansruedi Glatt [1,*] and Fabian Schumacher [1,2]

1 Department of Nutritional Toxicology, German Institute of Human Nutrition (DIfE) Potsdam-Rehbrücke, Arthur-Scheunert-Allee 114-116, 14558 Nuthetal, Germany; glatt@dife.de
2 Current address: Institute of Pharmacy, Freie Universität Berlin, Königin-Luise-Straße 2+4, 14195 Berlin, Germany; fabian.schumacher@fu-berlin.de
* Correspondence: glatt@dife.de; Tel.: +49-30-6916846

S1. Mutagenicity of NI3C in the *Salmonella typhimurium* strain TA100-hSULT1C2

S. typhimurium TA100-hSULT1C2 is engineered for the expression of human (h) sulphotransferase (SULT) 1C2, as described in [1]. It is important to know that we used the designation introduced by Freimuth *et al.* [2] in the strain name. Based on sequence similarities with SULT1C forms from other species, the initial designations for human SULT1C1 and SULT1C2 were later changed to SULT1C2 and SULT1C4, respectively, by Blanchard *et al.* [3]. We did not change the strain name to remain consistent with our previous publications.

We conducted unusually many mutagenicity experiments with NI3C in TA100-hSULT1C2, as this compound was used in the establishment of this strain. All results are presented in Figure S1. After transfection of the SULT1C2 expression vector into *S. typhimurium* TA100, NI3C was one of the first compounds tested in this strain (K1). In the first experiment (Exp. 1 in Figure S2), the highest increase in the number of revertant colonies was observed at the lowest NI3C dose used, 0.3 nmol (52 ng) per plate. At higher doses, the mutagenicity was masked by bacteriotoxicity, as manifested by a decrease in the number of revertant colonies and thinning of the his$^-$ background lawn. Subsequently we conducted three follow-up experiments in K1 with the inclusion of lower NI3C doses (Figure 2, top panel). In all experiments, the strongest increase in revertant colonies was detected at 0.1-0.3 nmol (17-52 ng) NI3C per plate. However, the number of spontaneous revertant colonies was relatively high (~200) in all four experiments conducted with the initial strain (K1) (Figure S2, top panel). We suspected that a spontaneous reversion might have occurred early during the expansion of K1, leading to an increased number of pre-existing revertants in our stock culture. For their elimination, we grew the strain at clonal densities on agar plates (medium supplemented with histidine and biotin). Then, individual colonies were expanded and used in mutagenicity experiments of NI3C and our standard SULT-dependent positive control, 1-hydroxymethylpyrene (1-HMP). The results for NI3C in these clones are presented in the middle panel of Figure S2. Clone C was excluded because of the very low number of spontaneous revertant colonies and low effects of NI3C and 1-HMP. The other four clones produced numbers of spontaneous revertant colonies (~100) similar to those found with the parental strain TA100. NI3C and 1-HMP showed clear mutagenicity, with less than 2-fold variation among the clones. We selected clone D, as it showed the lowest spontaneous revertant numbers, the strongest response to 1-HMP and a strong response to NI3C (after subtraction of the negative control about equal to that of clone A) – although all these differences among the clones might be within the intra-experimental variation of this assay. The bottom panel of Figure S2 shows the result of a further experiment with clone D, using a wide range of NI3C concentrations. These findings demonstrate that NI3C exhibits clear mutagenicity in all experiments conducted – the maximal increase in the revertant frequency consistently occurred at a dose level of 0.1-0.3 nmol (17-52 ng) NI3C per plate.

All our work with TA100-hSULT1C2 published in previous articles was carried out with clone D.

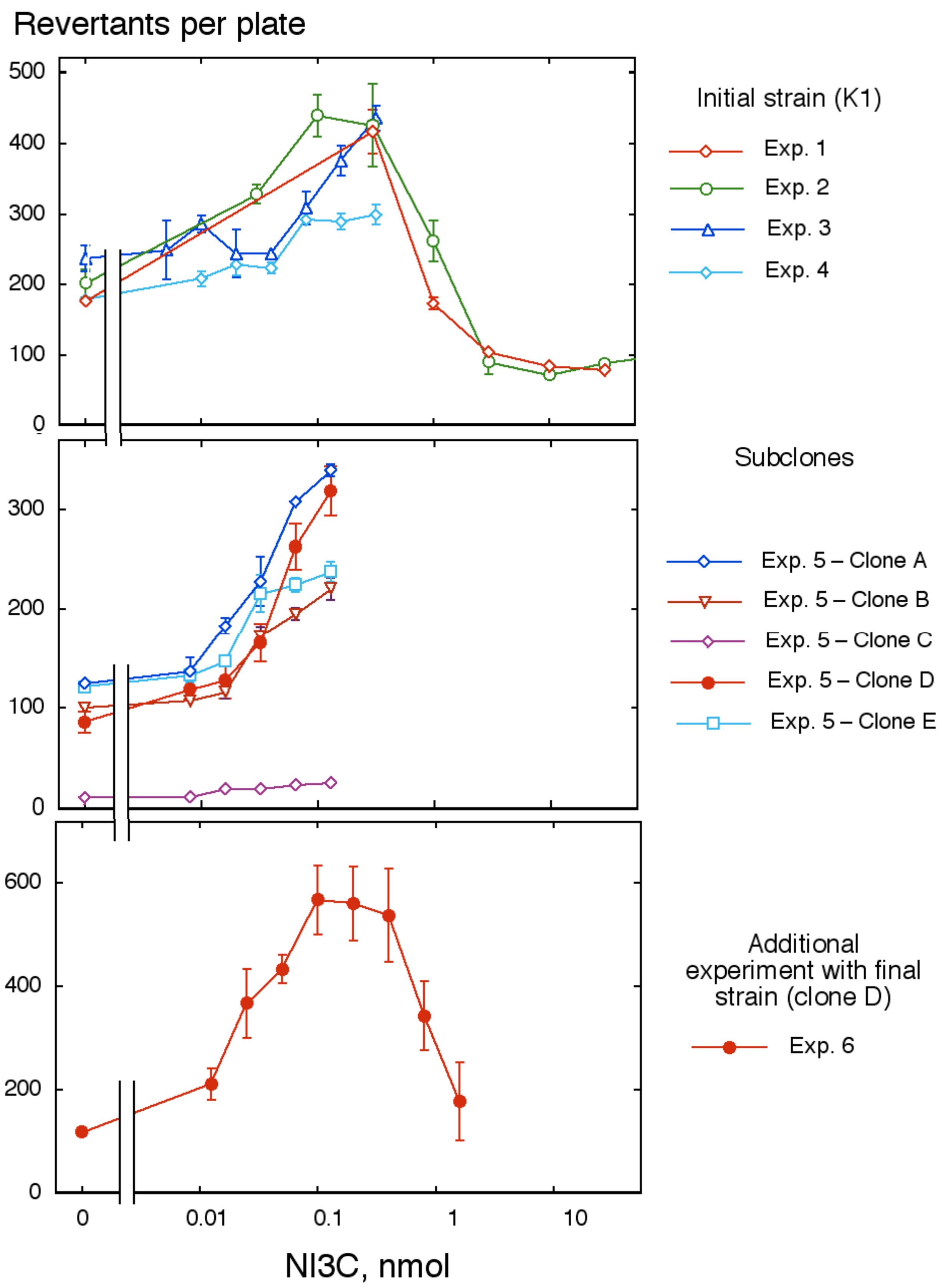

Figure S1. Mutagenicity of *N*-methoxyindole-3-carbinol (NI3C, upper panel) in the *S. typhimurium* TA100-hSULT1C2. The figure shows the results of all experiments of NI3C performed in this strain. Values are means ± SE of 3 plates (treatment groups) or 6 plates (vehicle controls).

S2. Specificity of the anti-hSULT1A1 antibodies used for immunostaining

As shown in Figure S2, kidney tissue from a control (wild-type) mouse remained completely unstained, under conditions that produced strong immunostaining with the anti-hSULT1A1 antibodies in mice carrying a human *SULT1A1-SULT1A2* transgene. This finding proves that the staining is attributable to the presence of human SULT1A1/2 protein produced from the transgene.

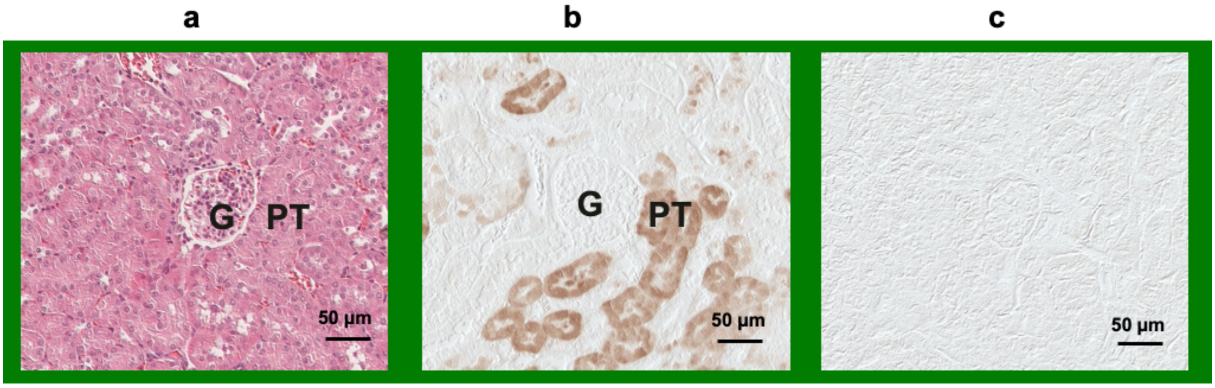

Figure S2. Immunohistochemical localisation of human SULT1A1/2 protein in the kidney of an FVB/N-hSULT1A1/2 mouse. Sections from kidney tissue were prepared and stained as described in section 4.8 of the actual publication. The kidneys were taken from 8-week old male FVB/N (wild-type) and FVB/N-hSULT1A1/2 mice, not treated with any test compounds.

a, b serial kidney sections from the FVB/N-hSULT1A1/2 mouse, stained with haematoxylin/eosin (a) or immunostained using an antiserum raised against human SULT1A1 (b)

c kidney sections from the wild-type mouse, immunostained as in panel b

G glomerulus

PT proximal tubule

S3. Design of animal experiment

The histological findings presented in this study were obtained with leftovers of tissue samples from a study with a different study aim. It was performed with permission (LUGV V3-2347-28-2011) of the Landesamt für Umwelt, Gesundheit und Verbraucherschutz of the State of Brandenburg, Germany. Key information:

S3.1 Animal strain and sex: Male hemizygous FVB/N-SULT1A1/2 mice. Homozygous FVB/N-SULT1A1/2 mice were constructed and propagated in our laboratory [4]. For the experiments, we used F1 animals obtained by breeding homozygous FVB/N-SULT1A1/2 mice with wild-type FVB/N mice.

S3.2 Maintenance conditions: The animals were maintained under specific pathogen-free, temperature- and light (12 h / 12 h)-controlled conditions. Body weights were determined daily. The staff handling the animals had a list of symptoms that required interventions (further observation, inspection by veterinarian, or immediate euthanasia).

S3.3 Number of animals: 30 mice treated with NI3C, 21 vehicle controls.

S3.4 Treatments: The mice received varying numbers (1–40) of oral treatments (gavage) with NI3C [150 µmol (26 mg) per kg body mass per treatment] or the vehicle only (glyceryl trioctanoate, 1.7 ml/kg body mass), three times per week (always on Monday, Wednesday and Friday), starting at the age of five weeks. Number of treatments with NI3C: 1 (3 mice: adducts), 2 (3 mice: adducts), 4 (3 mice: adducts), 10 (6 mice: adducts, global gene expression), 20 (3 mice: adducts), 40 (12 mice: adducts, global gene expression, organ weights, histology). Number of vehicle-control animals: 10 treatments (6 mice: adducts, global gene expression), 20 (3 mice: adducts), 40 (12 mice: adducts, global gene expression, organ weights, histology). Twenty-four hours after the last treatments, animals were anesthetized with isoflurane, and blood was taken by puncture of the retrobulbar venous plexus into heparinized tubes. Then, the animals were killed by cervical dislocation, and the organs were immediately frozen in liquid nitrogen.

S3.5 Endpoints (with key aspects)
- Body masses (daily), organ masses (terminal): paired Student's t test (if distribution is normal) or Mann–Whitney U test (otherwise) of NI3C-treated versus corresponding control mice
- Adducts: LC-MS/MS [5, 6]: one-way ANOVA with Bonferroni's with correction for multiple comparisons (if distribution is normal) or Kruskal–Wallis test (otherwise) for the time-course in NI3C-treated animals; no adducts expected to occur in Vehicle-controls.
- Gene expression: microarray and real-time RT-PCR analyses (Mann–Whitney U test)
- Histology: stomach, small intestine, colon, liver, lung, kidney. Statistics depending on endpoint (and compatibility with normal distribution): variance analysis, Student's t test; or Kruskal–Wallis test, or Mann–Whitney U test.

S3.6 Statistics: statistical tests as indicated in section 3.5; the single animals were used as statistical units; $p < 0.05$ was used as the criterion for statistical significance.

S3.7 Randomisation: Average body masses were similar for NI3C-treated and control animals (as well as the subgroups). The treatment sequence was not regulated *a priori*, as all treatments were very short (gavage, body mass determination, killing).

S3.8 Exclusion of animals from the evaluation: one vehicle-control animal had to be euthanised due to bad health state in week 6 of the experiment. All other animals showed no symptoms requiring intervention – no exclusions had to be made from any evaluations. The NI3C treatment had no influence on body mass throughout the entire treatment period.

S4. SCE frequencies in negative and positive controls

We present the SCE frequencies of negative and positive controls of all experiments conducted in our laboratory, if they met the following criteria:
- The cell lines used were V79 (sub-strain V79-MZ) or V79-hSULT1A1 (clone 5).
- All evaluations were conducted using encoded slides. This criterion led to the exclusion of the initial nine experiments.
- Two negative control cultures were used, and the SCE frequency was studied in 25 metaphases per culture. This criterion led to the exclusion of one experiment with V79 cells involving a single negative control (although the SCE frequency was in the usual range).
- The solvent was dimethylsulphoxide (20 µl per 5-ml culture), water (or PBS or medium, up to 100 µl per 5-ml culture). This criterion led to the exclusion of a few experiments involving other solvents (tetrahydrofuran, acetone, or ethanol). Note: the use of 20 µl ethanol consistently led to a statistically significant increase in the SCE frequency

The following characteristics were not used for the selection, but were met by all experiments selected:
- The exposure time was 30-32 h.
- Complete medium was used during the exposure period.
- External activating system: none or purified myrosinase.

The results of the negative controls are summarised in Table S1. In the opinion of an expert group [7], a 2-fold increase in the SCE frequency is a strong positive result with a test compound. Table S2 lists compounds that led to ≥ 2-fold increases in the SCE frequency in our work with V79-MZ and V79-hSULT1A1, together with the concentrations required (and used).

Many of these compounds were utilised as positive control compounds. Glycidamide was particularly often used as directly acting genotoxicant in V79-MZ cells as well as various V79-derived cell lines engineered for expression of some xenobiotic-metabolising enzymes. It consistently produced a strong effect in all experiments conducted.

Other compounds listed in Table 2 required the expression of SULT1A1 for strong SCE induction. These compounds are highlighted by red lettering. 1-Hydroxymethylpyrene was frequently used as a SULT-dependent positive control substance in V79-hSULT1A1 cells. It was also activated by various other SULT forms.

In the present study, individual experiments were conducted with V79p and V79-hCYP1A2-hSULT1C2 cells, lines we had only rarely used in earlier SCE tests. However, we had used V79p cells in many Hprt gene mutation tests – we never observed any difference in response compared to V79-MZ cells. And 1-hydroxymethylpyrene proved to be a suitable positive control in the SCE test in V79-hCYP1A2-hSULT1C2 cells (Table 1 in the main part of this study)

Table S1. SCE frequency of the negative controls of cell lines V79-MZ and V79-hSULT1A1 [1].

	V79-MZ	V79-hSULT1A1
Number of experiments	41	26 [2]
Number of SCE per metaphase		
Mean	6.94	6.84
SD	1.09	0.79
Highest value	8.87	8.96
Lowest value	4.15	5.35

[1] Each experiment contained two negative control cultures. The mean value of these cultures was used as the statistical unit.
[2] One additional experiment was conducted, but excluded, since the SCE frequency of its negative control was an outlier, exceeding the mean by > 5 * SD.

Table S2. Compounds that increased the SCE frequency strongly (by a factor of ≥ 2) in our experiments. [1]

V79-MZ	V79-hSULT1A1
Glycidamide, 100-400 μM [2]	Glycidamide, 100 μM
anti-Chrysene-1,2-dihydrodiol-3,4-oxide, 1 μM [2]	
1-Sulfooxymethylpyrene, 0.3 μM [2]	
Illudin S, 0.003-0.03 μM [2]	Illudin S, 0.003-0.03 μM
(-)-Acylfulvene, 3 μM [2]	(-)-Acylfulvene, 3 μM
	(-)-6-Hydroxyacylfulvene, 3 μM
2-Chloroallylic alcohol, 100-300 μM [2]	
Cyclophosphamide, 2000 μM [2]	
	1-Hydroxymethylpyrene, 0.3-3 μM [4]
	Neoglucobrassicin, 1-10 μM [3,4]
	N-Methoxyindole-3-carbinol (NI3C), 1-3 μM [4]
	(-)-1'-Hydroxymethyleugenol, 10-30 μM [4]
	(+)-1'-Hydroxymethyleugenol, 10-30 μM [4]
	(*E*)-3'-Hydroxymethylisoeugenol, 30-100 μM [4]

[1] Concentrations are given that led to ≥ 2-fold increases in the SCE frequency. All these statistically highly significant ($p < 10^{-5}$).
[2] Also tested in V79-derived cell lines engineered for expression of certain xenobiotic-metabolising enzymes with results similar to those observed in the parental cell line V79-MZ.
[3] Tested in the presence of myrosinase.
[4] No effect, or a much weaker effect, was observed in V79-MZ control cells. However, some compounds also showed strong effects in V79-derived cell lines engineered for expression of certain other SULT forms.

References

1. Meinl, W.; Pabel, U.; Osterloh-Quiroz, M.; Hengstler, J.G.; Glatt, H.R., Human sulfotransferases are involved in the activation of aristolochic acids and are expressed in renal target tissue. *Int. J. Cancer* **2006,** *118*, 1090-1097. doi: 10.1002/ijc.21480
2. Freimuth, R.R.; Raftogianis, R.B.; Wood, T.C.; Moon, E.; Kim, U.J.; Xu, J.; Siciliano, M.J.; Weinshilboum, R.M., Human sulfotransferases SULT1C1 and SULT1C2: cDNA characterization, gene cloning, and chromosomal localization. *Genomics* **2000,** *65*, 157-165. doi: 10.1006/geno.2000.6150
3. Blanchard, R.L.; Freimuth, R.R.; Buck, J.; Weinshilboum, R.M.; Coughtrie, M.H., A proposed nomenclature system for the cytosolic sulfotransferase (SULT) superfamily. *Pharmacogenetics* **2004,** *14*, 199-211. doi: 10.1097/00008571-200403000-00009
4. Dobbernack, G.; Meinl, W.; Schade, N.; Florian, S.; Wend, K.; Voigt, I.; Himmelbauer, H.; Gross, M.; Liehr, T.; Glatt, H.R., Altered tissue distribution of 2-amino-1-methyl-6-phenylimidazo[4,5-*b*]pyridine-DNA adducts in mice transgenic for human sulfotransferases 1A1 and 1A2. *Carcinogenesis* **2011,** *32*, 1734-1740. doi: 10.1093/carcin/bgr204
5. Barknowitz, G.; Engst, W.; Schmidt, S.; Bernau, M.; Monien, B.H.; Kramer, M.; Florian, S.; Glatt, H.R., Identification and quantification of protein adducts formed by metabolites of 1-methoxy-3-indolylmethyl glucosinolate *in vitro* and in mouse models. *Chem. Res. Toxicol.* **2014,** *27*, 188-199. doi: 10.1021/tx400277w
6. Schumacher, F.; Herrmann, K.; Florian, S.; Engst, W.; Glatt, H.R., Optimized enzymatic hydrolysis of DNA for LC-MS/MS analyses of adducts of 1-methoxy-3-indolylmethyl glucosinolate and methyleugenol. *Anal. Biochem.* **2013,** *434*, 4-11. doi: 10.1016/j.ab.2012.11.001
7. Tucker, J.D.; Auletta, A.; Cimino, M.C.; Dearfield, K.L.; Jacobson-Kram, D.; Tice, R.R.; Carrano, A.V., Sister-chromatid exchange: 2nd report of the Gene-Tox program. *Mutat. Res.* **1993,** *297*, 101-180. doi: 10.1016/0165-1110(93)90001-4
